# Immune complex handling in transplantation: central roles for complement factor H, animal models, and translational implications

**DOI:** 10.3389/fimmu.2026.1830799

**Published:** 2026-06-15

**Authors:** Richard J. Quigg, Jessy J. Alexander

**Affiliations:** Department of Medicine, SUNY University at Buffalo, Jacobs School of Medicine & Biomedical Sciences, Buffalo, NY, United States

**Keywords:** complement, factor H, immune complex, mouse model, transplant

## Abstract

Immune complexes (ICs) are increasingly recognized as dynamic regulators of graft injury in solid organ transplantation. Beyond their formation, the biological impact of ICs is determined by how they are handled, trafficked, and cleared. Complement plays a central role in this process, functioning not only as an effector system but as a context-dependent regulator of IC fate. Classical pathway activation initiates complement deposition on ICs, while the alternative pathway amplifies these signals, with regulatory proteins constraining excessive activation. Complement factor H (CFH), the principal regulator of the alternative pathway, emerges as a key determinant of IC handling by modulating complement amplification and directing ICs toward non-inflammatory clearance pathways. This review integrates mechanistic insights into IC biology with clinical observations across kidney, heart, and lung transplantation. We highlight species-specific differences in IC clearance, examine how complement-targeted therapies intersect with IC biology, and address ongoing controversies regarding complement as a marker versus driver of injury. Collectively, these concepts position IC handling, not merely for IC formation, but as a central determinant of transplant outcomes.

## 1 Introduction

Antibody-mediated rejection (AMR), as defined by Banff classification, remains a major barrier to durable allograft survival. Despite advances in immunosuppressive strategies targeting T cell activation, alloantibody formation persists (1), and donor-specific antibodies (DSA) drive Banff-defined active and chronic active AMR phenotypes (2), leading to microvascular inflammation, endothelial dysfunction, and progressive graft loss. These features are characteristic of antibody-driven rejection, whereas the etiology of microvascular inflammation in the absence of DSA or C4d remains incompletely understood. Central to these processes is the formation of antigen-antibody immune complexes (ICs) at the graft interface, which function not merely as intermediates of complement activation but as dynamic immunobiological platforms that integrate complement signaling, Fc receptor engagement, and cellular responses. While IC deposition within the allograft is a recognized feature of AMR, the contribution of circulating ICs in solid organ transplantation is less well established and is not typically considered a dominant driver of disease; this aspect will be further addressed in subsequent sections.

For decades, ICs in transplantation were viewed largely as passive intermediates whose primary relevance lay in their ability to activate complement, with C4d deposition serving as a durable diagnostic footprint (3, 4). However, this view fails to capture the dynamic nature of IC biology. Immune complexes vary widely in size, composition, immunoglobulin subclass, and complement opsonization state (5), all of which influence their inflammatory potential, cellular interactions, and tissue distribution. Importantly, the pathogenic impact of ICs is determined not only by their generation but also by the efficiency and route of their clearance.

**Figure 1:**
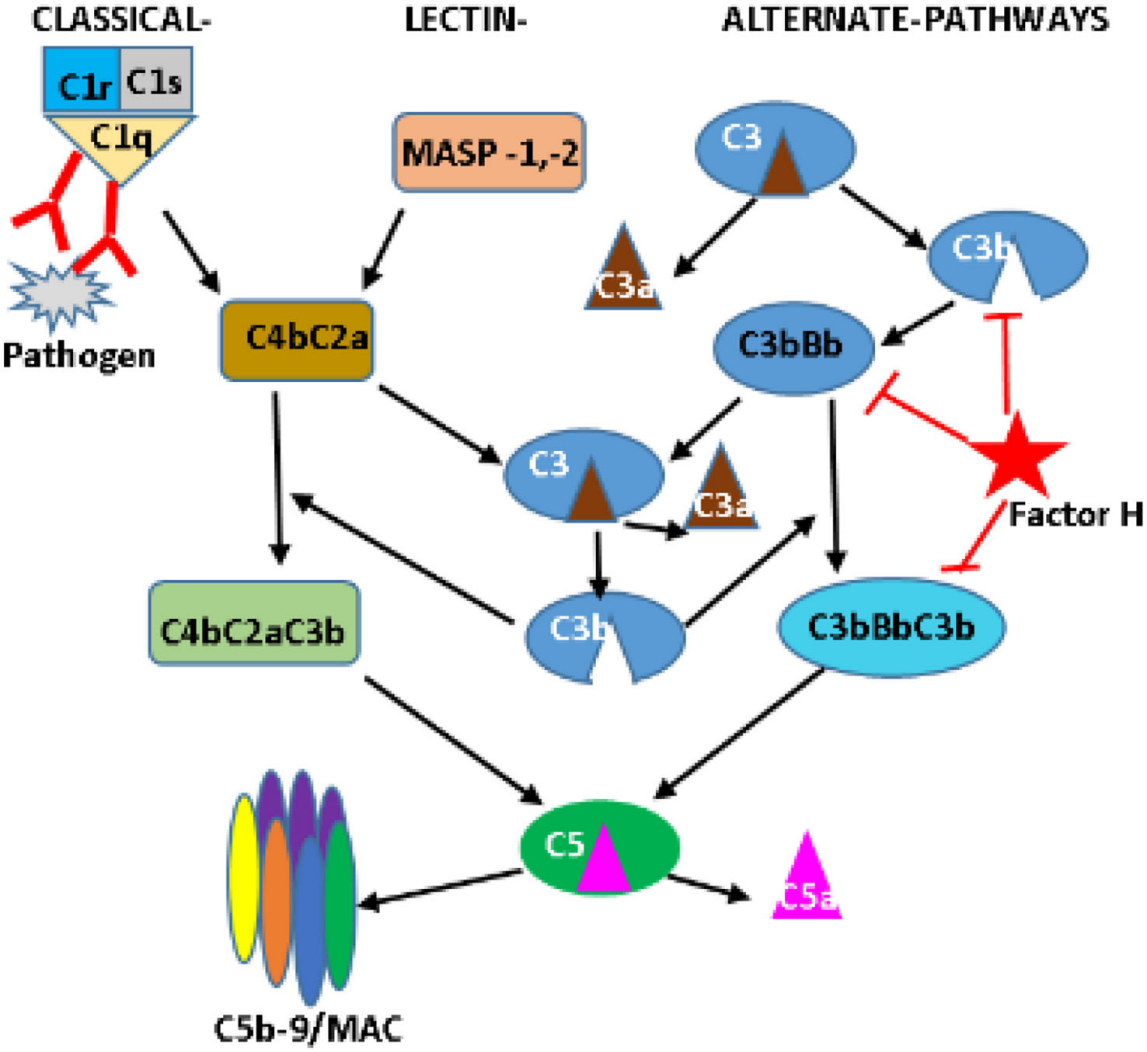
A schematic diagram of the complement system showing the three pathways, the classical, alternative and lectin pathways. The three pathways are initiated differently but end with the formation of the membrane attack complex. Shown in red is complement factor H (CFH) and the location of its action.

The complement system (**Figure 1**) lies at the intersection of IC formation, amplification, and resolution (6). While classical pathway activation initiates complement deposition on ICs, the alternative pathway provides powerful amplification that can dramatically escalate tissue injury (7). At the same time, complement regulators (8), particularly complement factor H (9), act as critical checkpoints that limit runaway activation and facilitate non-inflammatory IC handling (10). Insights from renal and vascular diseases have revealed that dysregulation of these regulatory pathways predisposes to IC-mediated pathology (11), as exemplified by conditions such as IgA Nephropathy (12) and C3 Glomerulopathy (13). In these disorders, IC formation is typically driven by intrinsic or systemic abnormalities, whereas in transplantation, AMR arises from alloantigen recognition and DSA formation, highlighting both shared effector mechanisms and distinct initiating triggers.

In this review, we advance a conceptual framework positioning IC handling, not just the IC formation, as a central determinant of transplant outcomes. We integrate mechanistic data with clinical observations across kidney, heart, and lung transplantation, emphasize species-specific differences that complicate preclinical modeling, and examine how emerging complement-targeted therapies may reshape transplant immunology.

## 2 Immune complex formation in transplantation

Immune complexes are positioned as the central organizing principle of antibody-mediated injury in transplantation, with complement serving as a key modulatory system that shapes downstream biological outcomes. Within this system, inhibitory regulators such as Complement factor H (9, 14) (CFH) play a critical role in limiting alternative pathway amplification, alongside other regulators including C4-binding protein (C4BP), membrane cofactor protein (CD46), and decay-accelerating factor (CD55) (8, 15), which collectively constrain complement activation across pathways. This framing shifts emphasis away from isolated complement readouts toward a systems-level understanding of how immune complexes are formed, modified, trafficked, and ultimately resolved or perpetuated within transplanted organs. Complement activation in this context reflects a dynamic interplay between the classical pathway (CP), typically initiated by antibody-antigen interactions, and the alternative pathway (AP), which serves as an amplification loop. The strength and quality of complement activation are influenced by the initiating trigger, antibody characteristics, and regulatory capacity, with mechanisms such as C2 bypass further illustrating cross-talk between pathways. Immune complexes arise whenever recipient antibodies encounter donor-derived antigens (16). In transplantation, this process is continuous rather than episodic, reflecting ongoing antigen exposure from viable graft tissue. The persistence of antigen distinguishes transplant-associated ICs from those formed during transient infections and places unique demands on regulatory pathways that must balance immune surveillance with tissue preservation. Similar conditions of sustained antigen exposure are also observed in autoimmune diseases, where dysregulation of IC handling and complement control contributes to chronic tissue injury, highlighting shared pressures on regulatory systems despite distinct initiating triggers.

While complement-dependent cytotoxicity (CDC) contributes to graft injury (17), it represents one of several effector mechanisms. Increasing evidence supports a prominent role for natural killer cells in antibody-mediated rejection (18), acting through antibody-dependent cellular cytotoxicity (ADCC) and “missing-self” recognition, particularly in later stages of transplantation. In addition, direct endothelial cell activation by antibodies further contributes to graft dysfunction (19). These mechanisms operate alongside complement activation, providing a more nuanced view of antibody-mediated injury beyond CDC alone.

### 2.1 Sources and molecular characteristics of immune complexes

In transplantation, ICs arise predominantly from interactions between recipient antibodies and donor-derived antigens. The most extensively studied antibodies are IgG DSA directed against HLA class I and II molecules expressed on graft endothelium (19). Beyond HLA-directed responses, a growing body of evidence implicates non-HLA antibodies targeting endothelial antigens (e.g., angiotensin II type 1 receptor), extracellular matrix components, and cryptic neoantigens exposed during ischemia-reperfusion injury (20) contribute substantially to IC formation (20). Additional sources include apoptotic and necrotic cell debris released during early graft injury that provide a continuous source of autoantigenic material capable of forming circulating and tissue-deposited ICs. Emerging evidence also implicates ICs formed against viral antigens (e.g., CMV, BK virus) and damage-associated molecular patterns, particularly in immunosuppressed transplant recipients, further expanding the antigenic landscape. The biological behavior of immune complexes is profoundly influenced by their molecular characteristics. Antibody subclass determines complement-fixing capacity (21), with IgG1 and IgG3 being potent activators of the classical pathway, whereas IgG2 and IgG4 exhibit more limited activity. Glycosylation patterns of IgG Fc regions further modulate complement engagement and Fc receptor signaling, adding an additional regulatory layer. Antigen density and valency influence IC size and avidity, which in turn affect tissue deposition and cellular uptake (22). In kidney transplantation, IgA-containing ICs may also emerge, particularly in recurrent glomerular diseases, adding further complexity to IC handling.

### 2.2 Immune complexes as active effectors of graft injury

Immune complexes exert pathogenic effects through multiple, intersecting mechanisms. First, they initiate complement activation (23), generating anaphylatoxins (C3a, C5a) that recruit and activate leukocytes and promote endothelial activation (24). Second, ICs engage Fc receptors on myeloid cells, platelets, and endothelial cells, serving a dual role by both mediating Fcγ receptor-dependent clearance of ICs by myeloid cells and, when this system is saturated or dysregulated, amplifying inflammatory signaling and cytokine production (25). Third, persistent IC deposition alters endothelial phenotype (26, 27), promoting procoagulant, pro-fibrotic, and proliferative responses that underlie chronic allograft vasculopathy and transplant glomerulopathy (28). Critically, impaired Fcγ receptor-mediated clearance, together with insufficient complement regulation, sustains these pathways, creating a feed-forward loop in which complement activation begets further IC deposition and tissue injury. This dynamic underscores the importance of regulatory mechanisms that constrain IC persistence.

### 2.3 The complement system as amplifier and regulator of immune complexes

In the context of ICs, complement should be conceptualized less as a linear effector cascade (**Figure 1**) and more as a regulatory decision system that determines whether immune complexes are resolved or perpetuated. While complement enhances clearance and solubilization of immune complexes, excessive activation drives inflammation, endothelial injury, and tissue damage. Classical pathway activation represents the initial sensing mechanism triggered by antibody-antigen engagement. However, it is the alternative pathway that functions as the dominant amplification loop, rapidly escalating complement activation once C3b is deposited on immune complex surfaces. Key differences in immune complex handling between humans and rodents are summarized in **Table 1**, while complement-targeted therapeutic strategies relevant to transplantation and immune complex biology are outlined in **Table 2**.

**Table 1:** Key differences in immune complex handling between humans and rodents.

Feature	Humans/primates	Rodents
Primary immune complex carrier	Erythrocytes	Platelets and platelet-derived microparticles
Dominant receptor/regulator	CR1 (CD35) on erythrocytes, supported by CFH	Platelet-associated complement regulators including CFH
Clearance site	Liver and spleen macrophages	Reticuloendothelial system with platelet involvement
Inflammatory potential	Low; non-inflammatory stripping of ICs	Higher; platelet activation and leukocyte recruitment
Translational implication	Efficient IC clearance with limited tissue deposition	Exaggerated inflammatory phenotypes and altered therapeutic responses

**Table 2:** Complement-targeted therapies relevant to transplantation and immune complex biology.

Therapeutic class	Target	Primary effect on IC biology	Potential limitation
C5 inhibitors	Terminal pathway	Reduce C5a and MAC-mediated injury downstream of ICs	Do not address upstream IC persistence or amplification
C3 inhibitors	Central complement hub	Broad suppression of IC opsonization and amplification	Risk of impaired IC clearance and infection
Alternative pathway inhibitors	Factor B/factor D	Limit amplification while preserving classical initiation	May not correct defective clearance pathways
CFH-based or CFH-enhancing strategies	Regulatory axis	Restore physiological IC regulation and clearance	Clinical development still emerging

This amplification confers both protective and pathological consequences, the complement functioning as a regulatory decision system determining whether immune complexes are resolved or perpetuated. On one hand, in physiological settings complement opsonization enhances solubilization of immune complexes, promotes their recognition by complement receptors, and facilitates efficient, non-inflammatory clearance via erythrocytes (in humans) (29) or platelet-associated pathways (in rodents) (30) (**Table 1**). In this context, complement serves as a homeostatic mechanism preserving tissue integrity. Conversely, excessive or dysregulated complement activation transforms immune complexes into potent inflammatory platforms. High-density deposition of C3 fragments and generation of anaphylatoxins (C3a, C5a) amplify leukocyte recruitment, endothelial activation, and cytokine production (31–33). Terminal pathway activation further contributes to membrane injury and procoagulant phenotypes (34, 35). Thus, complement operates as a context-dependent modulator, with its net effect determined by the balance between amplification and regulatory control. Importantly, the alternative pathway amplification loop represents a critical inflection point. Under physiological conditions, regulatory proteins tightly constrain this loop. However, in states of regulatory insufficiency, amplification becomes self-sustaining, driving persistent immune complex deposition and chronic tissue injury.

### 2.4 Complement factor H as the gatekeeper of immune complex fate

Complement factor H occupies a central position within this regulatory architecture and can be viewed as a gatekeeper that determines IC fate (36). CFH is the principal soluble regulator of the alternative pathway and functions not only as an inhibitor but as a surface-selective regulator that discriminates between host and non-host environments. By integrating surface recognition, convertase regulation, and opsonin processing, CFH directs complement activity toward controlled, non-inflammatory outcomes.

Mechanistically, CFH limits alternative pathway amplification by accelerating decay of the C3bBb convertase (37, 38) and by limiting factor B binding to C3b. It also serves as a cofactor for factor I-mediated cleavage of C3b into iC3b and C3dg, thereby shaping the nature of C3 fragment deposition and influencing downstream recognition and clearance pathways (39, 40). In addition, CFH binds polyanionic host surfaces and extracellular matrix components (41), thereby restricting complement activation to self-associated environments and promoting regulated handling of complement-opsonized immune complexes.

**Figure 2:**
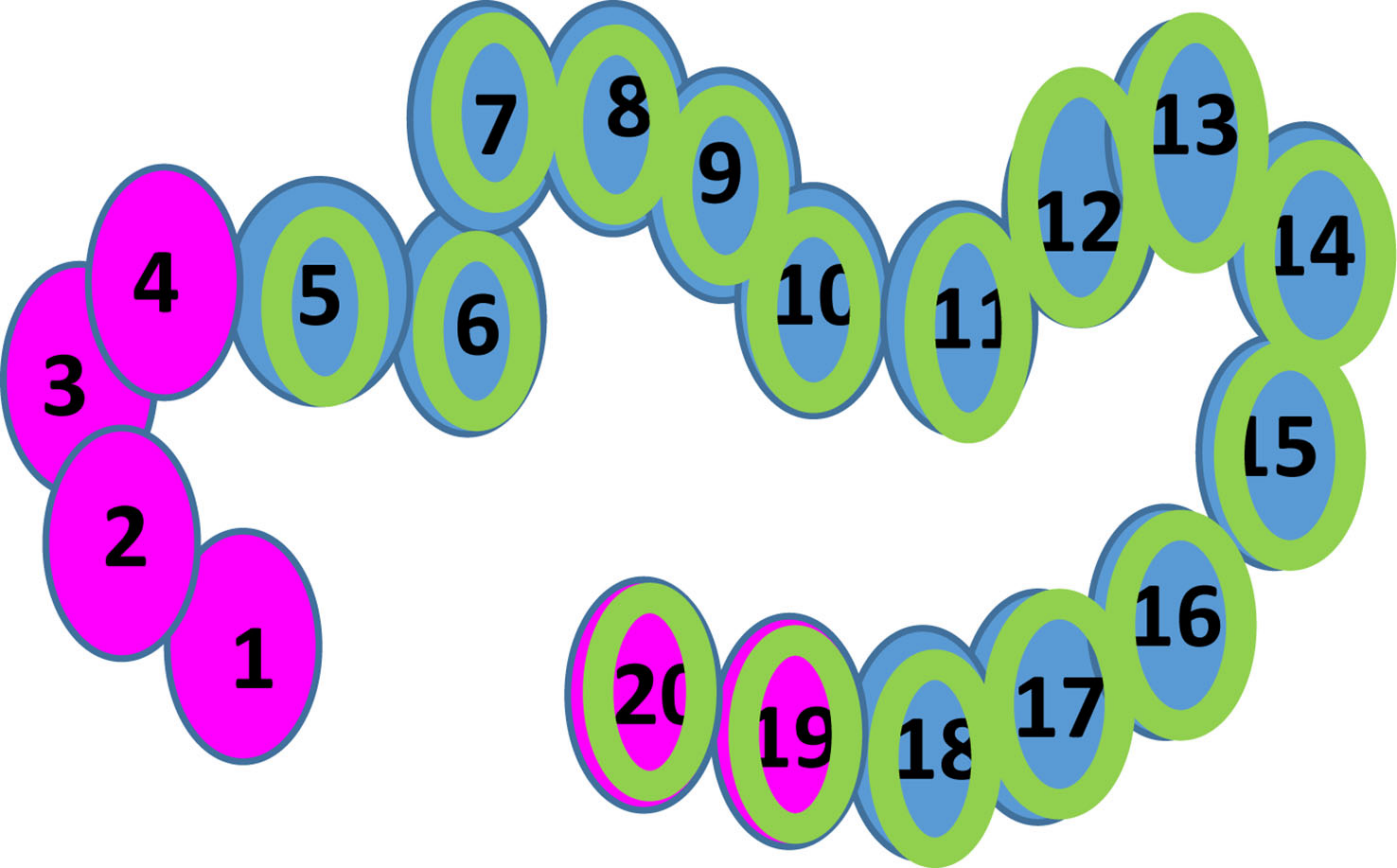
A schematic showing CFH with 20 short consensus repeats (SCRs) like beads on a chain. Each SCR is comprised of 60 amino acids joined by disulfide bonds. SCRs shaded in pink indicate C3b-binding regions or signify GAG/sialic acid-recognition sites (especially 7 and 20).

Selective molecular features underlie these functions. CFH consists of 20 short consensus repeat (SCR/CCP) domains, with CCP1–4 mediating regulatory activity (decay acceleration and cofactor activity) while CCP6–8 and CCP19-20) contribute to binding C3b/C3d in cooperation with host-associated polyanions (**Figure 2**) (42, 43). This modular architecture allows CFH to distinguish host-bound complement deposits, including C3-opsonized ICs from foreign surfaces, a distinction that is critical in transplantation where donor tissue must be protected despite ongoing antibody exposure. Importantly, CFH does not bind immune complexes directly but instead regulates complement activity on C3-decorated surfaces in a context-dependent manner.

Dysfunction of CFH due to genetic variation, autoantibodies, or inflammatory microenvironments (44) shift immune complex handling from controlled resolution toward persistence and excessive complement activation. This principle is well illustrated in C3 glomerulopathy (C3G) (45, 46), which reflects dysregulation of the alternative pathway due to impaired CFH function rather than classical antibody-driven IC disease (45) and is characterized by uncontrolled C3 deposition. Genetic variants in CFH and CFH-related proteins (47, 48), as well as acquired anti-CFH autoantibodies, underscore the pathological consequences of impaired regulation and are associated with diseases such as C3G and atypical hemolytic uremic syndrome. Experimental and clinical observations further demonstrate that local inflammatory conditions can overwhelm CFH regulatory activity, even in the absence of systemic deficiency. Collectively, these findings including studies such as studies such as Parente et al. (49) support the concept that CFH is not merely inhibitory but actively governs immune complex fate through regulation of complement amplification and deposition.

### 2.5 Immune complex clearance as an active biological process

Clearance of immune complexes should be understood as an active, regulated biological process rather than a passive consequence of complement opsonization. The nature of complement fragments deposited on immune complexes determines which cellular receptors are engaged and whether clearance occurs in a silent or inflammatory manner. CFH-mediated regulation is therefore not ancillary but foundational to immune complex biology in transplantation.

### 2.6 Classical and alternative pathway interplay

Complement activation on immune complexes is initiated primarily through the classical pathway via C1q binding (50). Subsequent complement amplification is largely driven by the alternative pathway, which, once C3b is deposited, rapidly amplifies opsonization and increases C3 fragment density on immune complexes, thereby enhancing their inflammatory potential. This amplification step is not intrinsically pathological but represents a physiological mechanism for signal amplification and efficient clearance. Dysregulation of this process due to genetic variation, acquired autoantibodies, or local inflammatory microenvironments shifts the balance toward excessive complement activation, sustained opsonization, and tissue injury.

## 3 Immune complex clearance pathways: humans versus rodents

**Figure 3:**
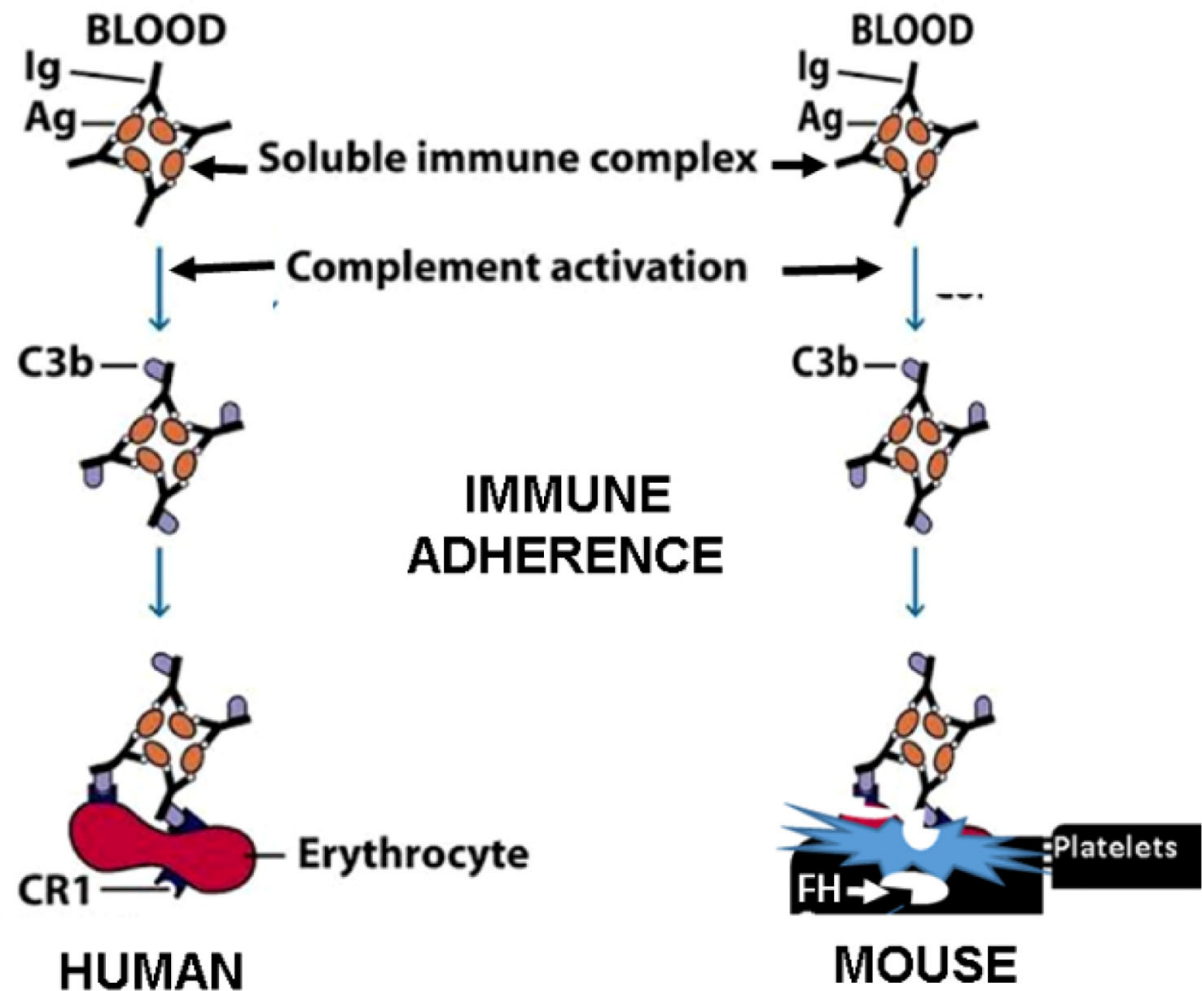
Immune adherence in man and mouse. The diagram shows immune adherence in man and mouse with RBCs and CR1 playing roles in humans that are replaced by platelets and CFH in mice.

In humans and other primates, erythrocytes play a central role in immune complex clearance through expression of complement receptor 1 (CR1/CD35) (**Figure 3**) (51). CR1 binds C3b- and C4b-opsonized immune complexes, transporting them to the liver and spleen for removal by macrophages (52, 53). This process allows efficient clearance without triggering inflammation or destroying the carrier erythrocyte. CFH indirectly supports this pathway by regulating the density and form of C3 fragments on immune complexes, thereby influencing CR1 engagement. Although this pathway primarily describes circulating immune complex handling, similar principles of complement opsonization and regulation are relevant to endothelial-bound immune complexes in transplantation, where local clearance and retention are governed by the same balance of complement activation and control. Variation in CR1 expression levels and CFH function may contribute to inter individual differences in IC handling and susceptibility to graft injury.

In rodents IC handling occurs via platelets with CFH acting as the immune adherence receptor (**Figure 3**) (30). Rodents lack erythrocyte CR1, relying instead on alternative mechanisms for IC handling (**Table 1**). In murine systems, platelets and platelet-derived microparticles bind complement-opsonized ICs, with CFH associating with activated platelets to regulate complement activation. This platelet-dependent pathway represents a functional replacement for erythrocyte-mediated clearance but introduces fundamentally different immunological consequences. Platelets actively participate in inflammation, leukocyte recruitment, and thrombosis (54), potentially exaggerating inflammatory phenotypes in rodent transplant models.

These fundamental differences necessitate caution when extrapolating findings from rodent transplant models to human clinical settings, particularly for therapies targeting complement or immune complex handling.

### 3.1 Organ-specific manifestations of immune complex injury

Kidney transplantation provides the most extensively characterized clinical context in which immune complex biology intersects with complement regulation (55). AMR, transplant glomerulopathy, and recurrent or *de novo* glomerular diseases all share a common denominator: sustained immune complex formation at the glomerular and peritubular capillary interface. Renal microvascular beds are uniquely vulnerable to immune complex deposition due to high blood flow, fenestrated endothelium, and abundant expression of complement-activating surfaces (56). Immune complexes (IC) deposited along the glomerular basement membrane or within mesangial regions initiate complement activation, but the severity of injury varies widely among patients with similar antibody burdens (57). This heterogeneity reflects not only differences in complement regulatory capacity, including CFH-mediated control of alternative pathway amplification, but also variation in antibody pathogenicity (e.g., IgG subclass and Fc properties) that influence complement activation and effector engagement. Clinically biopsies demonstrating heavy IgG and C3 deposition in the absence of crescents or sclerosis raise critical interpretive questions. Such findings may indicate effective containment of complement activation by intact regulatory pathways rather than absence of pathogenic potential. Conversely, progression to transplant glomerulopathy may represent failure of IC clearance rather than escalation of antibody production alone (58). Importantly, not all complement-driven renal pathologies are immune complex–mediated; for example, C3 glomerulopathy (45, 59) reflects primary alternative pathway dysregulation with dominant C3 deposition and may occur independently of classical antibody-driven immune complex formation. Complement deposition, particularly C4d and C3 fragments, correlates with microvascular inflammation and adverse outcomes (60). However, biopsies with heavy IgG and C3 deposition but limited structural injury highlight the complexity of IC pathogenicity and the importance of regulatory mechanisms.

In heart transplantation, IC-mediated injury manifests predominantly within the coronary microvasculature and contributes to the development of cardiac allograft vasculopathy (61). Unlike the kidney, where IC deposition is often directly visualized, cardiac pathology reflects the cumulative effects of IC driven endothelial activation, complement-mediated inflammation, and smooth muscle proliferation. Immune complexes engaging endothelial Fc receptors and complement receptors promote chronic endothelial dysfunction (62), creating a pro-inflammatory and pro-proliferative vascular environment. Complement activation fragments such as C3a and C5a amplify leukocyte recruitment and platelet activation (55, 63), linking IC biology to thrombo-inflammatory pathways in cardiac allografts. Immune complexes deposited within the coronary microvasculature activate complement, leading to generation of C3a and C5a, which recruit and activate neutrophils and monocytes (64). These cells, in turn, release proteases, reactive oxygen species, and pro-inflammatory cytokines that amplify endothelial injury. Regulation at the level of IC handling may therefore be a critical determinant of long-term cardiac graft survival. Simultaneously, complement activation promotes platelet activation and aggregation, bridging innate immunity and thrombosis. Platelets interacting with immune complexes via Fc receptors and complement receptors contribute to leukocyte recruitment and microvascular occlusion (65). Experimental studies have demonstrated that C5a signaling enhances platelet-leukocyte aggregate formation, a key feature of thrombo-inflammation (66). Endothelial cells themselves respond to immune complexes and complement fragments by adopting a procoagulant phenotype (67), characterized by upregulation of tissue factor, adhesion molecules, and reduced anticoagulant signaling. These processes collectively drive chronic vascular remodeling, smooth muscle proliferation, and luminal narrowing characteristic of cardiac allograft vasculopathy. Thus, immune complexes act as central nodes linking complement activation, endothelial dysfunction, platelet activation, and leukocyte recruitment into a unified thrombo-inflammatory axis that underlies chronic cardiac graft injury.

Lung transplantation presents a distinct immunological landscape in which immune complexes operate at the interface between alloimmunity and continuous environmental exposure (68). The lung’s vast surface area, resident immune cell populations, and exposure to inhaled antigens create conditions conducive to IC formation and persistence. Antibody-mediated rejection and chronic lung allograft dysfunction are increasingly recognized to involve IC-driven complement activation within the airway and microvascular compartments (69). In this setting, failure of IC clearance may be particularly deleterious, as ongoing epithelial injury amplifies antigen release and perpetuates immune activation. These features highlight the need for regulatory strategies that preserve immune defense while preventing immune complex-mediated tissue damage.

Lung transplantation presents a unique interface between alloimmunity and environmental exposure. Immune complexes contribute to antibody-mediated rejection and chronic lung allograft dysfunction, with complement activation amplifying epithelial and endothelial injury in a highly inflammatory milieu.

### 3.2 Treatment paradigms intersecting with immune complex biology

#### 3.2.1 Conventional therapies

Current therapies for AMR focus in addition to conventional immunosuppression, focus on antibody removal and immunomodulation, including plasmapheresis, intravenous immunoglobulin, and B cell directed therapies. While these approaches reduce circulating antibodies, they do not directly address complement amplification or IC clearance.

#### 3.2.2 Complement targeted therapeutics

Emerging complement inhibitors targeting C5, C3, factor B, and upstream components offer new opportunities to modulate IC-mediated injury (**Table 2**). However, broad inhibition may impair physiological IC clearance and host defense. Strategies that enhance CFH function or selectively modulate alternative pathway amplification may offer a more nuanced approach.

Positioning therapies within the framework of immune complex handling clarifies why some approaches attenuate injury transiently while others may offer more durable protection.

### 3.3 Controversies and knowledge gaps

*Is Complement a Marker or a Driver of Allograft Injury?* One of the most enduring debates in transplantation concerns whether complement activation represents a direct driver of tissue injury or a secondary marker of antibody engagement (70). Diagnostic reliance on complement deposition, particularly C4d, has reinforced the perception of complement as a surrogate of alloantibody activity rather than an independent pathogenic axis. However, this binary framing is challenged by observations such as C4d-negative antibody-mediated rejection, discordance between complement deposition and injury severity, and variable clinical responses to complement-targeted therapies, all of which indicate that complement activity does not uniformly track with disease expression. A more nuanced interpretation is that complement functions as a conditional driver whose pathogenic impact depends on the context of immune complex handling (71). In settings where immune complexes are efficiently regulated and cleared, complement deposition may be transient and clinically silent. Conversely, when regulatory pathways, most notably alternative pathway control is impaired, complement amplification sustains immune complex persistence and promotes tissue injury. Mechanistically, in acute antibody-mediated rejection, complement activation amplifies inflammation through generation of C3a and C5a, promoting leukocyte recruitment, endothelial activation, and microvascular injury; in this phase, complement inhibition can attenuate this amplification loop. In contrast, in chronic injury, structural remodeling, endothelial dysfunction, and smooth muscle proliferation become less dependent on ongoing complement activation, potentially limiting the efficacy of delayed complement blockade.

This context-dependent model is supported by clinical studies in which complement inhibition (e.g., targeting C5) has shown greater efficacy in early or acute antibody-mediated rejection compared with more modest or inconsistent effects in chronic transplant injury (72). Together, these observations suggest that complement may act as a driver in some contexts and a marker in others, depending on the timing, regulatory environment, and stage of disease.

*Timing, Duration, and Context of Complement Intervention:* Another area of active debate concerns the optimal timing and duration of complement-targeted therapies. Early intervention during acute antibody-mediated rejection may blunt inflammatory amplification, whereas delayed inhibition in established chronic injury may yield limited benefit. Importantly, indiscriminate or prolonged complement suppression raises concerns regarding host defense and physiological immune complex clearance. From an immune complex perspective, the effectiveness of complement inhibition may depend less on the specific target and more on whether the intervention restores regulatory balance. Therapies that constrain amplification while preserving clearance pathways may offer advantages over broad blockade strategies, particularly in chronic or smoldering disease states.

### 3.4 Interpreting immune complex and complement deposition in biopsies

The interpretation of graft biopsies containing immune complexes and complement deposition remains challenging. Heavy immunoglobulin and C3 deposition in the absence of crescents, sclerosis, or overt microvascular inflammation is frequently encountered and often generates clinical uncertainty. Importantly, complement deposition does not uniformly indicate injury; for example, persistent C4d positivity in the absence of rejection is well described in ABO-incompatible transplantation and is thought to reflect accommodation rather than ongoing tissue damage. Rather than representing benign findings, such patterns may reflect a dynamic equilibrium in which immune complexes are present but effectively regulated.

This perspective emphasizes the importance of integrating histologic findings with functional assessments of complement activity and immune complex regulation, rather than relying solely on static deposition patterns. Such assessments may include measurements of complement activation products (e.g., C3a, C5a, soluble C5b-9), pathway activity assays (classical and alternative pathway functional assays), evaluation of complement regulatory capacity, and analysis of opsonization patterns (e.g., C3 fragment profiling) that influence receptor engagement and clearance. Future diagnostic approaches may benefit from biomarkers that capture IC turnover and regulatory capacity.

### 3.5 Species differences and translational uncertainty

Finally, the reliance on rodent models continues to generate uncertainty regarding the translational relevance of complement-targeted interventions. Differences in IC handling mechanisms (30, 52), particularly the absence of erythrocyte CR1 and the prominence of platelet-centered pathways may skew inflammatory readouts and therapeutic responses. A measured interpretation of preclinical data that explicitly accounts for these differences is essential to avoid overgeneralization. Collectively, these controversies do not undermine the relevance of complement or immune complexes in transplantation but instead highlight the need for context-dependent interpretation. A directional shift toward understanding IC handling as a regulated biological process offers a unifying framework for resolving many of these debates and guiding future investigation.

## 4 Future directions and translational opportunities

Future investigation must move beyond static assessments of IC deposition toward dynamic evaluation of IC handling and regulation in transplant recipients. Integrating genetic variation in complement regulators, circulating biomarkers of complement activation, and functional assays of IC clearance will be essential for defining patient subsets most likely to benefit from targeted intervention. The development of humanized experimental systems, including ex vivo organ perfusion and erythrocyte reconstituted animal models, offers promising avenues to bridge current translational gaps.

## 5 Clinical translation and trial design implications

An IC centered framework provides important guidance for the clinical application and evaluation of complement-targeted therapies in transplantation. An immune complex–centered framework provides a structured basis for complement-targeted therapy in transplantation. Three core principles emerge. First, patient selection must be biology-driven rather than antibody- or deposition-driven. Therapeutic response depends on whether disease reflects active complement dysregulation (e.g., impaired alternative pathway control or defective CFH-mediated regulation) versus adequately regulated immune complex handling. Accordingly, stratification should incorporate biomarkers of complement activation, regulatory capacity, and immune complex turnover rather than relying on antibody burden or static tissue deposition alone.

Second, timing is critical. Complement inhibition is most effective when applied during early antibody-mediated injury, where it can interrupt amplification loops and promote resolution, whereas in established chronic injury dominated by fibrosis and vascular remodeling, benefit is likely limited. Third, therapeutic strategy should prioritize restoration of regulation rather than broad complement suppression. Approaches that reinforce endogenous regulatory pathways, including CFH-dependent mechanisms, may provide more durable control while preserving host defense.

## 6 Conclusions

IC handling represents a central, underappreciated determinant of transplant outcomes across organ systems. Rather than acting solely as passive byproducts or diagnostic markers, immune complexes function as dynamic immunobiological entities whose fate is governed by complement amplification, regulatory checkpoints, and clearance pathways. Complement factor H occupies a pivotal position at this intersection, shaping whether immune complexes are silently resolved or perpetuate inflammation, endothelial injury, and chronic graft dysfunction.

A unifying theme emerging from this synthesis is that dysregulation of IC handling, rather than IC formation alone helps explain key clinical paradoxes in transplantation, including the dissociation between antibody burden and injury severity, the variable significance of complement deposition, and the limited durability of existing therapies. Species-specific differences in IC clearance, particularly the reliance on erythrocyte CR1 in humans versus platelet dependent mechanisms in rodents, further underscore the need for caution in translational inference and therapeutic development.

In summary, immune complexes are not passive byproducts of alloimmunity but dynamic platforms whose fate is actively determined by complement regulation and clearance mechanisms. Future therapeutic strategies should focus on restoring regulatory balance rather than eliminating complement activity. Complement factor H occupies a central position in this regulatory network, shaping whether ICs are resolved or drive persistent inflammation and chronic graft injury. A shift toward understanding IC handling as a regulated biological process provides a unifying framework for interpreting clinical heterogeneity and for guiding the development of next-generation complement-targeted therapies.
